# Clinical value and cost analysis of the sFlt-1/PlGF ratio in addition to the spot urine protein/creatinine ratio in women with suspected pre-eclampsia: PREPARE cohort study

**DOI:** 10.1186/s12884-022-05254-1

**Published:** 2022-12-06

**Authors:** M. Wind, M. E. van den Akker-van Marle, B. E. P. B. Ballieux, C. M. Cobbaert, T. J. Rabelink, J. M. M. van Lith, Y. K. O. Teng, M. Sueters

**Affiliations:** 1 Department of Obstetrics, Leiden University Medical Centre, P.O. Box 9600, 2300 RC Leiden, the Netherlands; 2 Department of Biomedical Data Sciences, Leiden University Medical Centre, Leiden, the Netherlands; 3 Department of Clinical Chemistry, Leiden University Medical Centre, Leiden, the Netherlands; 4 Department of Nephrology, Leiden University Medical Centre, Leiden, the Netherlands

**Keywords:** Pre-eclampsia, Protein/creatinine ratio, sFlt-1/PlGF, Telemonitoring, Cost analysis

## Abstract

**Background:**

This study investigated the clinical value of adding the sFlt-1/PlGF ratio to the spot urine protein/creatinine ratio (PCr) in women with suspected pre-eclampsia.

**Methods:**

This was a prospective cohort study performed in a tertiary referral centre. Based on the combination of PCr (< 30) and sFlt-1/PlGF (≤38) results, four groups were described: a double negative result, group A−/−; a negative PCr and positive sFlt-1/PlGF, group B−/+; a positive PCr and negative sFlt-1/PlGF, group C+/−; and a double positive result, group D+/+. The primary outcome was the proportion of false negatives of the combined tests in comparison with PCr alone in the first week after baseline. Secondary, a cost analysis comparing the costs and savings of adding the sFlt-1/PlGF ratio was performed for different follow-up scenarios.

**Results:**

A total of 199 women were included. Pre-eclampsia in the first week was observed in 2 women (2%) in group A−/−, 12 (26%) in group B−/+, 4 (27%) in group C+/−, and 12 (92%) in group D+/+. The proportion of false negatives of 8.2% [95% CI 4.9–13.3] with the PCr alone was significantly reduced to 1.6% [0.4–5.7] by adding a negative sFlt-1/PlGF ratio. Furthermore, the addition of the sFlt-1/PlGF ratio to the spot urine PCr, with telemonitoring of women at risk, could result in a reduction of 41% admissions and 36% outpatient visits, leading to a cost reduction of €46,- per patient.

**Conclusions:**

Implementation of the sFlt-1/PlGF ratio in addition to the spot urine PCr, may lead to improved selection of women at low risk and a reduction of hospital care for women with suspected pre-eclampsia.

**Trial registration:**

Netherlands Trial Register (NL8308).

**Supplementary Information:**

The online version contains supplementary material available at 10.1186/s12884-022-05254-1.

## Background

The accuracy of current diagnostic assessments, blood pressure measurements and the spot urine protein/creatinine ratio (PCr), in predicting which women will develop pre-eclampsia and related adverse outcomes is poor [1–5]. In recent studies, the determination of soluble FMS-like tyrosine kinase-1 (sFlt-1) and placental growth factor (PlGF) has shown potential value for predicting the absence of pre-eclampsia [6–10]. The PROGNOSIS study showed that a sFlt-1/PlGF ratio of ≤38 had a negative predictive value of 99.3% for ruling out development of pre-eclampsia within the next week in women with suspected pre-eclampsia [6]. Ruling out the development of pre-eclampsia for a certain time period may lead to a reduction in over-diagnosis, redundant admission and outpatient visits, over-treatment and will consequently lower the costs [11–13].

Notwithstanding the impressive test characteristics, it remains matter of debate whether the introduction of this novel test can indeed translate to a reduction in pre-eclampsia-related hospital admissions and healthcare costs. Several studies have shown (INSPIRE, PARROT-UK, PARROT-Ireland) that hospital admissions and/or complication rate are essentially unchanged despite the improved prediction and selection of women with suspected pre-eclampsia [14–16]. This could possibly be explained by the fact that adding the sFlt-1/PlGF ratio to clinical practice including PCr for promoting patient safety (i.e. reduce false negatives), will also inevitably lead to more positive results, potentially causing an increase in health care usage rather than a reduction.

On the other hand, since the COVID-19 pandemic, in-hospital blood pressure monitoring in routine antenatal care has been rapidly shifting to self-monitoring at patients’ home. A recent study has shown that a clinical pathway with telemonitoring for women at risk of pre-eclampsia allows fewer antenatal visits and admissions, with no differences in perinatal outcomes [17, 18]. Therefore, we hypothesized that a better selection of women at risk for complications, by combining the sFlt-1/PlGF ratio and PCr, should be accompanied with de-escalation of care in the form of telemonitoring for those women identified to be at intermediate risk for development of pre-eclampsia and complications.

The present study investigated the potential value of the sFlt-1/PlGF ratio in addition to the spot urine PCr for predicting pre-eclampsia. The primary goal was to formulate a clinical prediction rule, combining the PCr and the sFlt-1/PlGF ratio in women with suspected pre-eclampsia, to rule out pre-eclampsia more safely in a large proportion of women. Secondary, in order to reduce the need for hospitalization and de-escalate the care we suggest a novel indication, based on the clinical prediction rule, for telemonitoring of women at risk.

## Methods

### Study design and population

The PREPARE study (PREdiction of Pre-eclampsia and AdveRse Events) was a prospective cohort study in a third line obstetrical care, medical centre (Leiden University Medical Centre (LUMC)). Both spot urine PCr and sFlt-1/PlGF sampling was performed in all pregnant women presenting with pre-eclampsia symptoms between December 2017 and February 2020. After the clinician specified the reason for suspicion of pre-eclampsia, 20 mL blood was sampled by venepuncture which was stored at an independent laboratory at − 80 °C. Analysis of the biomarkers was performed on the fully automated Elecsys® system (Cobas® analyzers, Roche Diagnostics International Ltd.) after study completion. As clinicians were unaware of the angiogenic factor levels, all women received follow-up and treatment according to local protocol following usual care where decision to monitor or admit a patient is based mainly on clinical manifestation, routine screening laboratory results in combination with spot urine PCr results [19]. Inclusion criteria were maternal age ≥ 16 years, singleton gestation, gestational age of ≥20 and < 37 weeks (based on sonography in the first trimester). Exclusion criterium was pre-eclampsia diagnosis before baseline day. The study was registered in the Netherlands Trial Register (NL8308) and approval for the study was obtained by the Medical Ethical Committee of the LUMC. Written informed consent was obtained from all women.

### Outcomes

The goal of the study was to determine the potential of the sFlt-1/PlGF ratio in addition to the spot urine PCr in ruling out pre-eclampsia for 1 week, with the proportion of false negatives as the main indicator for test safety. Therefore, the primary outcome was the proportion of false negatives of the combined tests in comparison with PCr alone in the first week after baseline. Secondary outcomes were the occurrence of adverse maternal/perinatal outcomes in the first week after baseline, including a combined endpoint of any pre-eclampsia, adverse maternal or perinatal outcome. Furthermore, we determined consequential costs, and “correct” hospital admissions defined below.

In order to assess the additional value of the sFlt-1/PlGF ratio, we compared usual care with a test scenario which subdivided the baseline groups by PCr with cut-off < 30 (mg/mmol) and the sFlt-1/PlGF ratio with cut-off ≤38 for ruling out pre-eclampsia during the first week after baseline. Four groups were described: a double negative result, group A−/−; a negative PCr and positive sFlt-1/PlGF ratio, group B−/+; a positive PCr and negative sFlt-1/PlGF ratio, group C+/−; and a double positive result, group D+/+. For the clinical prediction rule, a double negative result (group A−/−) was considered negative, while any positive result was considered positive (group B−/+, C+/−, and D+/+).

### Definitions

Suspected pre-eclampsia was defined as one or more of following symptoms identified by the clinician: new onset of elevated blood pressure (systolic blood pressure ≥ 140 mmHg and/or a diastolic blood pressure ≥ 90 mmHg) or proteinuria (positive dipstick or PCr ≥30 performed earlier), aggravation of pre-existing hypertension or proteinuria, epigastric pain, excessive oedema, headache, visual disturbances, sudden weight gain, low platelets (< 150 × 10^9^/L), elevated liver transaminases (alanine aminotransferase (ALT) or aspartate aminotransferase (AST) > 40 IU/L) or suspicion of fetal growth restriction (FGR, estimated fetal weight < 10th centile [20]). Significant proteinuria was defined as 24-hour collection ≥300 mg/day, or in absence of a 24-hour measurement PCr ≥30 [21]. Pre-eclampsia and gestational hypertension were defined according to 2018 guidelines of the International Society for the Study of Hypertension in Pregnancy (ISSHP) [21].

Maternal adverse outcomes were defined in line with full PIERS including death, stroke, eclampsia, blindness, uncontrolled hypertension (requiring administration of three or more different parenteral antihypertensive agents within a 12 hour period), the use of inotropic agents, pulmonary oedema (diagnosed clinically with one/more of oxygen saturation < 95%, diuretic treatment or x-ray confirmation), respiratory failure (needing intubation), myocardial ischemia or infarction, hepatic dysfunction (leading to disseminated intravascular coagulation), hepatic hematoma or rupture (confirmed by imaging or at laparotomy), renal failure (serum creatinine > 200 μmol/L), and transfusion of any blood products [22]. Other adverse outcomes were hypertension requiring administration of intravenous antihypertensives, thromboembolic events (arterial, venous or small vessel thrombosis, other than superficial venous thrombosis, in any tissue or organ), and perinatal adverse outcomes: preterm delivery (spontaneous and iatrogenic before 37 and 32 weeks), fetal growth restriction, admission to the neonatal intensive care-unit (NICU) and perinatal death.

Additional health care use consisted of hospital admissions, home monitoring and extra visits to the outpatient clinic including diagnostic tests during the first week after baseline. Health care use was scored “additional” if, from the patients’ record, it was clearly driven by (suspected) pre-eclampsia or pre-eclampsia in differential diagnosis and it did not fit in the usual care antenatal visit schedule. Home monitoring is a relocated hospital admission, comprising daily antenatal visits of dedicated nurses at patients’ home, including clinical assessment, cardiotocography and blood pressure measurement. Telemonitoring is a digital platform enabling home blood pressure measurements and pre-eclampsia symptoms reporting, in line with the SAFE@HOME study [17]. In order to assess whether admissions could be safely reduced, admissions were retrospectively scored “correct” if pre-eclampsia or any related adverse outcome was diagnosed in 1 week after baseline.

### Cost analysis

An explorative cost analysis was performed from a health care perspective comparing the cost of usual care with the cost of the test scenario. For this evaluation we compared usual care costs to theoretical costs in the test scenario where decision on follow-up will be made on both PCr and sFlt-1/PlGF result. Based on our results we assessed two theoretical scenarios in which the following assumptions were made:

Scenario 1: both tests negative (A−/−), no additional health care use in the following week; one of both tests positive (B−/+ and C+/−), one extra outpatient visit the next week and admissions conform usual care; both tests positive (D+/+), direct admission of all women. No women will be assigned to home monitoring.

Scenario 2: both tests negative (A−/−), no additional health care use in the following week; one of both tests positive (B−/+ and C+/−), telemonitoring during the next week and “correct” admissions conform usual care; both tests positive (D+/+), direct admission of all women. In scenario 2 we assumed that by implementing telemonitoring for the next week, including daily home blood pressure measurements and symptom questionnaires, women will only be admitted if they develop pre-eclampsia and no women will be assigned to home monitoring.

Costs included intervention costs and health care use. Intervention costs are the additional costs of the sFlt-1/PIGF test. These were assumed to amount 80,- euro per test [11, 12]. Costs of health care use were obtained by multiplying the additional health care use consisting of hospital admissions, home monitoring and extra visits to the outpatient clinic including diagnostic tests during the first week after baseline from the patients’ records with their cost prices. Cost prices of hospital days, outpatient visits, nursing time for home monitoring, interventions and diagnostics performed were valued using Dutch references costs, gross salaries of nurses, and tariffs for diagnostics of the Dutch Healthcare Authority [23]. Cost of telemonitoring was based on data from the SAFE@HOME study, were a prospective group of pregnant women at risk of pre-eclampsia used a digital health platform in a novel care pathway [18]. The general Dutch consumer price index was used to convert costs to 2020 price levels [24]. Timeframe of the cost analysis was until 1 week after baseline visit per patient. See supporting information for a detailed description of health care costs.

### Sample size and statistical analysis

Sample size calculations were based on studies on the accuracy of the spot urine PCr and sFlt-1/PlGF ratio to rule out respectively significant proteinuria (≥0.3 g/24 h) and pre-eclampsia [6, 25]. PCr with cut-off 30 has a false negative rate of approximately 13% in ruling out significant proteinuria and the sFlt-1/PlGF ratio with cut-off 38 has a false negative rate of 12–20% (1 - sensitivity 80–88%) for ruling out pre-eclampsia within 1 week [6, 25]. For power analysis, we hypothesized that application of the combination of both PCr and sFlt-1/PlGF ratio has clinical benefit if a false negative rate of less than 5% could be achieved. In order to show a hypothesized reduction of 13% false negatives to 5%, together with a pre-eclampsia prevalence of 25% in our study population, a sample size of approximately 150 women was needed (α = 5%; power = 80%).

Baseline characteristics and outcomes were summarized within four groups based on PCr result combined with sFlt-1/PlGF ratio as these tests will be presented simultaneously to the physician in daily clinical practice. Descriptive statistics are reported as frequency (%) or median ± interquartile range (IQR) depending on data type. False negatives and false positives were calculated as percentages with 95% confidence intervals (CI) and to assess statistical significance a z-test for two population proportions (α = 0.05) was used. For the analysis of data Statistical Package for the Social Sciences (SPSS) version 25.0 was used.

## Results

### Baseline characteristics

**Fig. 1:**
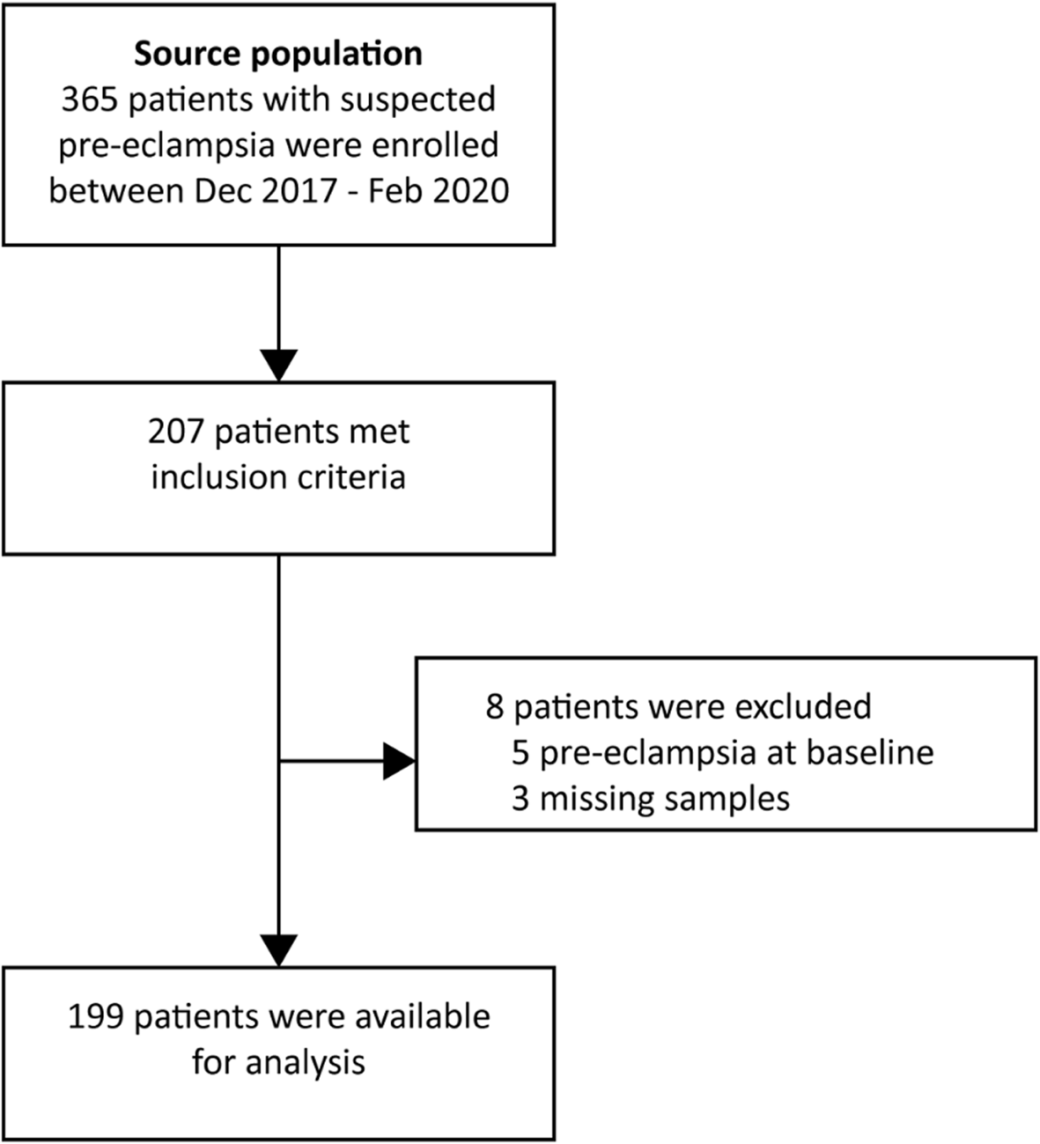
Flowchart inclusions

Between December 2017 and February 2020, 207 women met the inclusion criteria from the 365 women with suspected pre-eclampsia in the source population. After exclusion of 8 women, the analysis included 199 eligible participants (Fig. 1).

There were no withdrawals or losses to follow up. 124 women had a double negative result, group A−/−; 47 women a negative PCr and positive sFlt-1/PlGF ratio, group B−/+; 15 women a positive PCr and negative sFlt-1/PlGF ratio, group C+/−; and 13 women a double positive result, group D+/+.

Baseline characteristics for each cohort including reason for inclusion are shown in Table 1. In the groups with negative sFlt-1/PlGF ratio at baseline, A−/− and C+/−, new onset hypertension and low estimated fetal birth weight were less often reason for inclusion, respectively 36 and 40%, compared to those with a positive ratio, B−/+ and D+/+, 68 and 77%. Five of the women in group B−/+ (11%) were included for suspicion of isolated FGR. Vice versa, headache and visual disturbances were seen more often in the groups with negative sFlt-1/PlGF ratio at baseline. Interestingly, only 17 out of the 25 (68.0%) 24-hour urine samples collected directly after positive PCr testing at baseline, showed significant proteinuria above 0.3 g/24 h. This was most noteworthy in group C+/− with a median PCr of 42 (32–76) and 43% of the following 24-hour urine samples showing significant proteinuria.

**Table 1:** Baseline characteristics of each cohort

*Patients (N)*	Total *N = 199*	Group A−/− *N = 124*	Group B−/+ *N = 47*	Group C+/− *N = 15*	Group D+/+ *N = 13*
**General characteristics**					
Maternal age ^a^	32 (28–35)	31 (28–34)	32 (28–35)	32 (29–33)	32 (27–36)
Gestational age ^a^	33.5 (29.2–35.6)	32.6 (28.4–35.5)	34.6 (31.2–36.2)	33.5 (31.3–35.0)	36.0 (31.2–36.4)
Caucasian	183 (92.0)	115 (92.7)	45 (95.7)	12 (80.0)	11 (84.6)
Smoking during pregnancy	12 (6.0)	6 (4.8)	5 (10.6)	1 (6.7)	0
BMI (kg/m^2^) ^a^	26.5 (23.4–31.4)	27.6 (23.8–31.3)	24.4 (22.2–30.1)	30.1 (22.8–33.2)	27.7 (22.3–31.5)
**Reason of inclusion** ^**b**^					
New onset of hypertension	93 (46.7)	45 (36.3)	32 (68.1)	6 (40.0)	10 (76.9)
Aggravation of pre-existing hypertension	24 (12.1)	16 (12.9)	4 (8.5)	3 (20.0)	1 (7.7)
New onset of proteinuria	11 (5.5)	3 (2.4)	0	5 (33.3)	3 (23.1)
Aggravation pre-existing proteinuria	2 (1.0)	0	0	2 (13.3)	0
Epigastric pain	45 (22.6)	33 (26.6)	8 (17.0)	3 (20.0)	1 (7.7)
Headache	102 (51.3)	71 (57.3)	16 (34.0)	10 (66.7)	5 (38.5)
Excessive oedema	11 (5.5)	6 (4.8)	4 (8.5)	1 (6.7)	0
Visual disturbances	36 (18.1)	25 (20.2)	6 (12.8)	5 (33.3)	0
Sudden weight gain	2 (1.0)	0	2 (4.3)	0	0
Haemolysis	2 (1.0)	0	1 (2.1)	0	1 (7.7)
Elevated liver transaminases	8 (4.0)	5 (4.0)	2 (4.3)	0	1 (7.7)
Low platelets	7 (3.5)	3 (2.4)	4 (8.5)	0	0
Estimated Fetal Weight < 10th centile	29 (14.6)	8 (6.5)	14 (29.8)	1 (6.7)	6 (46.2)
**Gestational characteristics**					
Nulliparous	87 (43.7)	47 (37.9)	25 (53.2)	6 (40.0)	9 (69.2)
History of pre-eclampsia	35 (17.6)	25 (20.2)	7 (14.9)	3 (20.0)	0
Gestational diabetes	18 (9.0)	10 (8.1)	5 (10.6)	2 (13.3)	1 (7.7)
**Medical history**					
Chronic hypertension	28 (14.1)	22 (17.7)	3 (6.4)	2 (13.3)	1 (7.7)
Diabetes mellitus	5 (2.5)	4 (3.2)	1 (2.1)	0	0
Thromboembolic events	7 (3.5)	5 (4.0)	1 (2.1)	1 (6.7)	0
Renal disease	7 (3.5)	3 (2.4)	1 (2.1)	3 (20.0)	0
**Medication at baseline**					
Aspirin prophylaxis	57 (28.6)	39 (31.5)	8 (17.0)	8 (53.3)	2 (15.4)
Antihypertensive agents	24 (12.1)	15 (12.1)	5 (10.6)	2 (13.3)	2 (15.4)
**Clinical characteristics**					
Systolic blood pressure (mmHg)	132 (125–145)	130 (120–140)	137 (127–145)	140 (120–145)	155 (143–163)
Diastolic blood pressure (mmHg)	88 (78–94)	85 (75–90)	90 (85–100)	80 (70–90)	100 (88–105)
Protein/creatinine ratio (mg/mmol)	15 (12–22)	13 (11–18)	16 (11–20)	42 (32–76)	51 (39–134)
≥ 0.3 g/24 h/collected following PCr	18/29 (62.1)	0/2	1/2 (50.0)	6/14 (42.9)	11/11 (100.0)
sFlt-1/PlGF ratio	14 (5–50)	7 (2–16)	81 (55–156)	10 (6–33)	144 (87–520)
sFlt-1 (pg/ml)	3195 (1957–6284)	2299 (1477–3257)	6885 (5765–9168)	4896 (2087–6017)	10,386 (8254–14,199)
PlGF (pg/ml)	206 (114–455)	322 (196–516)	81 (47–117)	253 (173–540)	75 (31–103)

Data depicted as numbers (%) unless otherwise specified. Group A−/− = PCr < 30 and sFlt-1/PlGF ≤38. Group B−/+ = PCr < 30 and sFlt-1/PlGF > 38. Group C+/− = PCr ≥30 and sFlt-1/PlGF ≤38. Group D+/+ = PCr ≥30 and sFlt-1/PlGF > 38. BMI = body Mass Index. ^a^ Median (IQR), ^b^Combination of reasons possible

### Main results

**Fig. 2:**
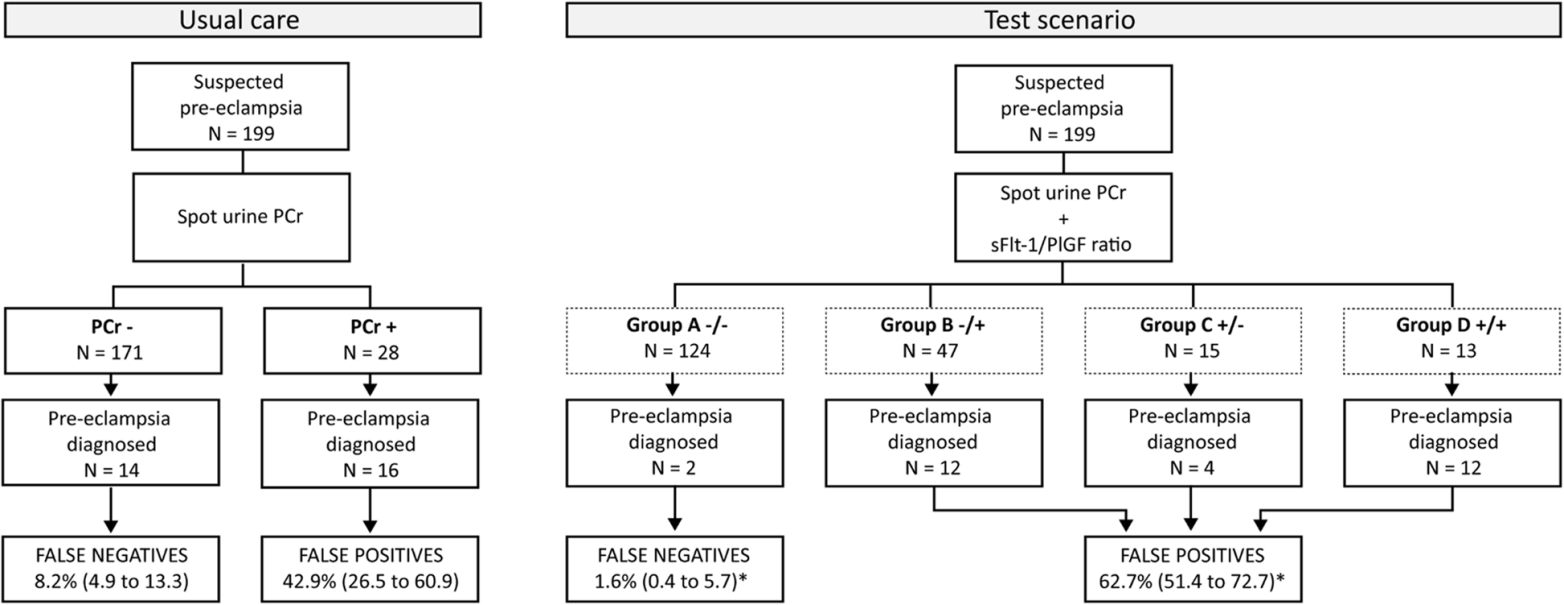
Pre-eclampsia diagnosis in the first week after baseline comparing usual care with test scenario. * Shows a significant difference with two-sided α < 0.0

As can be seen in Table 2, within 1 week after baseline, the primary endpoint pre-eclampsia was observed in 2 women (2%) of group A−/−, 12 (26%) in group B−/+, 4 (27%) in group C+/−, and 12 (92%) in group D+/+. When comparing usual care to the test scenario in Fig. 2, a proportion of false negatives of 8.2% [95% CI 4.9–13.3] was seen in the women with negative PCr at baseline in usual care, while a significant reduction of false negatives was seen in the test scenario when a negative sFlt-1/PlGF ratio was added, 1.6% [0.4–5.7]; (*p* = 0.01) in group A−/−. In addition, no maternal or perinatal adverse outcomes occurred in the first week after baseline when both tests were negative.

**Table 2:** Clinical outcomes in the first week after baseline

*Patients (N)*	Group A−/− *N = 124*	Group B−/+ *N = 47*	Group C+/− *N = 15*	Group D+/+ *N = 13*
Pre-eclampsia diagnosis	2 (1.6)	12 (25.5)	4 (26.7)	12 (92.3)
Maternal adverse outcome	0	1 (2.1)	1 (6.7)	4 (30.8)
Perinatal adverse outcome	0	3 (6.4)	0	7 (53.8)
**Combined endpoint** ^**a**^	**2 (1.6)**	**14 (29.7)**	**4 (26.7)**	**13 (100.0)**

Data depicted as numbers (%) unless otherwise specified. Group A−/− = PCr < 30 and sFlt-1/PlGF ≤38. Group B−/+ = PCr < 30 and sFlt-1/PlGF > 38. Group C+/− = PCr ≥30 and sFlt-1/PlGF ≤38. Group D+/+ = PCr ≥30 and sFlt-1/PlGF > 38

^a^Combined endpoint of patients with any pre-eclampsia, maternal or perinatal adverse outcome

### Health care usage and cost analysis

Table 3 describes the additional health care use in the first week after testing the 199 women: 29 (14.6%) were admitted, 63 (31.7%) women were seen for at least one additional visit at the outpatient clinic, and 8 (4.0%) were allocated to the home-monitoring program. Importantly, in 18/29 hospital admissions (62%) pre-eclampsia evolved to a necessary in-hospital intervention, whereas 11/29 admissions were not accompanied by the necessity of an intervention and potentially could have been avoided.

**Table 3:** Additional health care use in first week after baseline driven by (suspected) pre-eclampsia

*Patients (N)*	Group A−/− *N = 124*	Group B−/+ *N = 47*	Group C+/− *N = 15*	Group D+/+ *N = 13*
Admission	7 (5.6)	5 (10.6)	8 (53.3)	9 (69.2)
Mean (±SD) length of stay ^a^	1.6 ± 0.8	4.4 ± 2.8	3.1 ± 2.6	3.0 ± 1.7
Correct admission	1/7	4/5	4/8	9/9
Admission for immediate induction	1 (0.8)	2 (4.3)	0 (0.0)	4 (30.8)
Home monitoring	2 (1.6)	2 (4.3)	1 (6.7)	3 (23.1)
Correct home monitoring	0/2	2/2	1/1	3/3
Additional outpatient clinic visits	30 (24.2)	20 (42.6)	11 (73.3)	2 (15.4)
1 additional visit	21/30	14/20	7/11	1/2
2 additional visits	8/30	5/20	4/11	1/2
3 additional visits	1/30	1/20	0/11	0/2

Data depicted as numbers (%) unless otherwise specified. Group A−/− = PCr < 30 and sFlt-1/PlGF ≤38. Group B−/+ = PCr < 30 and sFlt-1/PlGF > 38. Group C+/− = PCr ≥30 and sFlt-1/PlGF ≤38. Group D+/+ = PCr ≥30 and sFlt-1/PlGF > 38. ^a^Mean length of stay with a maximum of 7 days used in calculations

In Table 4, the cost analysis for the test scenarios is described: scenario 1 showed similar costs compared to usual care implementing the sFlt-1/PlGF ratio in addition to the PCr. Although a potential reduction of 24% admissions, 100% home monitoring, and 42% outpatient visits could be achieved, the cost of the novel test will lead to a small or negligible reduction in costs compared to usual care. In scenario 2, with the assumption that the implementation of telemonitoring will lead to “correct” admissions, one could achieve a greater reduction in health care costs per patient. In this scenario, adding the sFlt-1/PlGF ratio to the spot urine PCr, with telemonitoring of the groups at risk, results in a potential reduction of 41% admissions, 100% home monitoring, and 36% visits to outpatient clinic during the first week after baseline. This could have consequentially led to a cost reduction of on average €46,- per patient.

**Table 4:** Cost analysis comparing usual care to theoretical test scenarios in the first week after baseline

*Health care use*	Prices (€)	Usual care (€)	Scenario 1 (€) ^a^	Difference 1 (€)	Scenario 2 (€)^a^	Difference 2 (€)
sFlt-1/PlGF ratio test	80	–	15,920	+ 15,920	15,920	+ 15,920
Admissions ^b^	71 + 590/day	52,227	45,120	− 7107	39,571	−12,656
Home monitoring ^c^	109 + 108/day	4752	0	− 4752	0	−4752
Additional outpatient clinic visits/Telemonitoring ^d^	168/visit 121/patient	14,279	8231	− 6047	6541	− 7738
Total per cohort	–	71,258	69,271	− 1987	62,032	− 9226
Total per patient	–	358	348	−10	312	−46

Data depicted in Euros (€)

^a^Scenario 1 = both tests negative (A−/−), no additional health care use in the following week; one of both tests positive (B−/+ and C+/−), one extra outpatient visit the next week and admissions conform usual care; both tests positive (D+/+), direct admission of all patients. Scenario 2: both tests negative (A−/−), no additional health care use in the following week; one of both tests positive (B−/+ and C+/−), telemonitoring during the next week and “correct” admissions conform usual care; both tests positive (D+/+), direct admission of all patients

^b^Mean length of stay was used for calculations. Calculations admissions usual care: 29 × 71 + 590x(7 × 1.6 + 5 × 4.4 + 8 × 3.1 + 9 × 3.0), Scenario 1: 22 × 71 + 590x(7 × 1.6 + 5 × 4.4 + 8 × 3.1 + 9 × 3.0). Scenario 2: 17 × 71 + 590x(4 × 4.5 + 4 × 5.0 + 9 × 3.0)

^c^Average number of days of home monitoring during the first week (4.5 days) was used. Calculations home monitoring usual care: 8 × 109 + 108 × 36

^d^Calculations outpatient visits usual care: 168 × 85. Scenario 1: 168 × 30. Scenario 2 (telemonitoring): 121 × 54

## Discussion

This study demonstrates that introduction of the sFlt-1/PlGF ratio on top off standard-of-care evaluation of the spot urine PCr has clinical value in the care for women with suspected pre-eclampsia. The proportion of false negatives of 2% when combining both tests was significantly improved compared to the 8% in the standard-of-care with urine PCr only. We modelled that with the combination of tests a relevant reduction in unnecessary admissions and outpatient visits could be achieved, and a cost reduction was attainable when non-invasive follow-up in the form of telemonitoring was added in women at intermediate risk with either a positive sFlt-1/PlGF ratio or urine PCr test.

The present study is unique in its design with direct evaluation of the additional value of the sFlt-1/PlGF ratio to usual care. Although the study was not powered for maternal or perinatal adverse outcomes, none were seen in the women with a PCr < 30 and sFlt-1/PlGF ratio ≤ 38 during the first week after baseline. A recent large retrospective real-world study evaluated the sFlt-1/PlGF ratio in a multimarker model including proteinuria on maternal and fetal adverse outcomes, which showed an area under the curve of 88.7% [26]. This study underlines the value of the combination of available clinical information with the sFlt-1/PlGF ratio to improve detection of adverse outcomes and our data further establishes evidence that the combination of both tests could contribute to improved precision in predicting pre-eclampsia.

Our study used the ISSHP 2018 definition for pre-eclampsia as the main outcome [21]. This could have led to lower sensitivity of the sFlt-1/PlGF ratio, as proteinuria was not obligatory for the diagnosis of pre-eclampsia and consequently women were diagnosed with hypertension in combination with e.g. laboratory abnormalities, persistent visual scotomas, or suspected FGR. Nonetheless, test characteristics were similar to previous studies, including high negative predictive value and area under the curve values for ruling out pre-eclampsia within 1 week with cut-off ≤38 [6, 9].

Previous studies have investigated the potential cost-savings of introducing the sFlt-1/PlGF ratio to rule out pre-eclampsia for which the budget impact analysis was mostly based on patient level data from the multinational PROGNOSIS study [11, 12, 27, 28]. The cost-savings in these studies were mostly based on hospitalization outcomes. We based our cost analysis in this study on self-collected clinical data and a new way of monitoring women with suspected pre-eclampsia, demonstrating not only a reduction in hospital admissions for women presenting with suspected pre-eclampsia but also a reduction on the frequency of visits to the outpatient clinic [11, 12, 28]. We identified groups B−/+ and C+/− as at intermediate risk with 27–30% pre-eclampsia development in the next week. Costs in these patients could be reduced when innovative telemonitoring can be employed as demonstrated in our explorative cost analysis. This form of self-monitoring in high-risk pregnant women is highly desirable since the COVID-19 pandemic and has already been established to contribute to reduced pre-eclampsia related admissions and visits to the outpatient clinic [17]. In the INSPIRE study, implementation of the sFlt-1/PlGF did not lead to reduced pre-eclampsia related admissions, probably caused by the high amount of false-positive results. Based on our study results, we believe that de-escalation of care, by implementing telemonitoring as follow-up for women at intermediate risk, could ultimately lead to the intended increase in cost-efficiency, without compromising patients’ safety. Further research should be performed to test this hypothesis [14].

The cost-savings presented in this study are representative for daily practice of Dutch healthcare, which in the Netherlands alone, with approximately 170,000 pregnancies per year, could lead to cost savings of 394,000-788,000,- euros a year. Since 5–10% of pregnancies is complicated by hypertensive disorders and even more women will present with symptoms contributing to suspected pre-eclampsia, implementing the sFlt-1/PlGF ratio will presumably lead to substantial change in antenatal care in the Netherlands [29].

Besides the important strengths of this study, our study also had its limitations. First, the sample size is relatively small and therefore underpowered to detect multiple adverse outcomes with low prevalence. Second, the spot urine PCr performed less accurate than hypothesized as 68.0% of the 24-hour urine samples collected directly after positive PCr testing at baseline, showed significant proteinuria above 0.3 g/24 h [25, 30]. This was the main reason for the relatively few pre-eclampsia diagnoses in group C+/− (26.7%), despite positive PCr testing, and may have led to over estimation of the effect of adding the sFlt-1/PlGF ratio in our study population. Third, Caucasian participants were overrepresented in this study, which should be considered when extrapolating these results into clinical practice. Another important limitation is the theoretical setting, in which we could only investigate the potential additional value of the test for the first week after baseline, instead of direct effect on allocation of health care, indicated deliveries, and costs. Hence, the result of false-positive tests and consequential uncertainty, extra visits or admissions could not be measured. Furthermore, for the cost analysis, calculations were made from a health care perspective, meaning patient perspectives such as productivity losses for paid and unpaid labour (such as care for other children and household activities) were not included. Also, only the effect on the costs for the first week after baseline could be calculated. This could have led to underestimation of the cost savings in our cohort, although potential re-testing was also excluded from the analysis.

## Conclusions

Based on the results of this study, we believe that the sFlt-1/PlGF ratio could be safely implemented in current standard-of-care. The costs of implementing this test might reduce when self-monitoring can be offered to women with suspected pre-eclampsia with either a negative sFlt-1/PlGF ratio or urine PCr test, potentially leading to more efficient allocation of health care and reduction of the burdensome hospital visits and admissions. Therefore, future research should be directed at combining the implemented sFlt-1/PlGF ratio with telemonitoring of women at intermediate risk (e.g. group B−/+ and C+/−). Moreover, the cost-effectiveness of implementing the sFlt-1/PlGF ratio within real-life clinical practice needs to be investigated in the Netherlands.

## Supplementary Information

**Additional file 1: Table S1.** Test characteristics combining PCr result with sFlt-1/PlGF ratio for pre-eclampsia diagnosis within one week after baseline. **Table S2.** Cost prices of health care use including diagnostics in 2020 euros. **Table S3.** Costs per group in the first week after baseline in usual care (in 2020 €). **Table S4.** Detailed outcomes within one week after baseline. **Table S5.** Overall pregnancy outcomes of each cohort.

## Data Availability

The datasets used and analyzed during the current study are available from the corresponding author on reasonable request.

## Abbreviations

PREPARE: PREdiction of Pre-eclampsia and AdveRse Events
LUMC: Leiden University Medical Centre
sFlt-1: Soluble FMS-like tyrosine kinase-1
PIGF: Placental growth factor
PCr: Spot urine protein/creatinine ratio
ALT: Alanine aminotransferase
AST: Aspartate aminotransferase
FGR: Fetal growth restriction
NICU: Neonatal intensive care-unit
ISSHP: International Society for the Study of Hypertension in Pregnancy
IQR: Interquartile range
CI: Confidence interval
SPSS: Statistical Package for the Social Sciences
