# Supplementary material for: Clinical value and cost analysis of the sFlt-1/PlGF ratio in addition to the spot urine protein/creatinine ratio in women with suspected pre-eclampsia: PREPARE cohort study
Source: BMC Pregnancy Childbirth. 2022 Dec 6;22:910. doi: 10.1186/s12884-022-05254-1 (PMC9727903; doi:10.1186/s12884-022-05254-1)
Supplement: Supplementary file 1 — Additional file 1: Table S1. Test characteristics combining PCr result with sFlt-1/PlGF ratio for pre-eclampsia diagnosis within one week after baseline. Table S2. Cost prices of health care use including diagnostics in 2020 euros. Table S3. Costs per group in the first week after baseline in usual care (in 2020 €). Table S4. Detailed outcomes within one week after baseline. Table S5. Overall pregnancy outcomes of each cohort. [file 12884_2022_5254_MOESM1_ESM.docx]

**Supporting information**

Table S1. Test characteristics combining PCr result with sFlt-1/PlGF ratio for pre-eclampsia diagnosis within one week after baseline.

| **Pre-eclampsia in one week** | **Usual care**  *(PCr only)* | **Test scenario**  *(A-/- vs. B-/+, C+/- and D+/+)* |
| --- | --- | --- |
| Sensitivity | 53.3 (35.8-70.4) | 93.3 (77.9-99.2) |
| Specificity | 92.9 (88.4-96.1) | 72.2 (64.8-78.8) |
| Negative predictive value | 91.8 (87.1-95.3) | 98.2 (93.4-99.5) |
| Positive predictive value | 57.1 (38.8-74.2) | 40.1 (34.0-46.5) |
| Likelihood ratio + | 7.5 (4.0-14.2) | 3.4 (2.6-4.4) |
| Likelihood ratio - | 0.5 (0.3-0.7) | 0.1 (0.0-0.4) |

Data presented as percentage (95% confidence interval). Group A-/- = PCr <30 and sFlt-1/PlGF ≤38. Group B-/+ = PCr <30 and sFlt-1/PlGF >38. Group C+/- = PCr ≥30 and sFlt-1/PlGF ≤38. Group D+/+ = PCr ≥30 and sFlt-1/PlGF >38.

Table S2. Cost prices of health care use including diagnostics in 2020 euros

| *Unit* | **Price (in 2020 Euros)** | **Source** ^1, 2, 3^ |
| --- | --- | --- |
| *Hospital admission* |  |  |
| Standard cost per day | 521.74 | Kostenhandleiding 2014 |
| Diagnostics per day (CTG, 6x blood pressure) | 68.47 | NZA |
| Diagnostics at admission (ultrasound, blood sampling, PCr) | 71.00 | NZA |
| *Home monitoring* |  |  |
| Standard cost per day | 107.84 | CAO UMC, NZA |
| At admission (outpatient clinic visit, PCr) | 108.71 | Kostenhandleiding 2014, NZA |
| *Visit to outpatient clinic* |  |  |
| Standard cost per visit | 167.99 | Kostenhandleiding 2014, CAO UMC, NZA |
| Telemonitoring | 121.13 | Heuvel et al. 2021 |

1. Kanters TA, Bouwmans CAM, van der Linden N, Tan SS, Hakkaart-van Roijen L. Update of the Dutch manual for costing studies in health care. PLoS One. 2017;12(11):e0187477.

2. Statistics Netherlands, Open Data [Internet]. 2020 [cited 11-5-2020]. Available from: <https://opendata.cbs.nl/#/CBS/nl/dataset/83131NED/table?ts=1604565807698>

3. van den Heuvel JFM, van Lieshout C, Franx A, Frederix G, Bekker MN. SAFE@HOME: Cost analysis of a new care pathway including a digital health platform for women at increased risk of pre-eclampsia. Pregnancy Hypertens. 2021 Jun;24:118-23.

Table S3. Costs per group in the first week after baseline in usual care (in 2020 €)

| *Patients (N)* | **Group A-/-**  *N = 124* | **Group B-/+**  *N = 47* | **Group C+/-**  *N = 15* | **Group D+/+**  *N = 13* |
| --- | --- | --- | --- | --- |
| Admission (x mean length of stay) | 7107 | 13340 | 15205 | 16575 |
| Home monitoring | 1188 | 1188 | 594 | 1782 |
| Additional outpatient clinic visits | 6719 | 4536 | 2520 | 504 |
| Total costs | 15015 | 19063 | 18319 | 18861 |

Group A-/- = PCr <30 and sFlt-1/PlGF ≤38. Group B-/+ = PCr <30 and sFlt-1/PlGF >38. Group C+/- = PCr ≥30 and sFlt-1/PlGF ≤38. Group D+/+ = PCr ≥30 and sFlt-1/PlGF

Table S4. Detailed outcomes within one week after baseline

| *Patients (N)* | **Group A -/-**  *N = 124* | **Group B -/+**  *N = 47* | **Group C +/-**  *N = 15* | **Group D +/+**  *N = 13* |
| --- | --- | --- | --- | --- |
| Preeclampsia diagnosis *(N)* | **2 (1.6)** | **12 (25.5)** | **4 (26.7)** | **12 (92.3)** |
| *Hypertension, accompanied with one or more:* |  |  |  |  |
| Proteinuria | 0 | 3/12 | 4/4 | 12/12 |
| Maternal organ dysfunction | 2/2 | 3/12 | 0 | 3/12 |
| Uteroplacental dysfunction | 0 | 6/12 | 1/4 | 5/12 |
| HELLP syndrome | 0 | 1/12 | 0 | 2/12 |
| Platelet count <150x10^9^/L | 0 | 6/12 | 0 | 2/12 |
| Elevated AST or ALT levels >40 U/L | 0 | 2/12 | 0 | 3/12 |
| Elevated LDH levels >250 U/L | 0 | 1/12 | 1/4 | 3/12 |
| Serum creatinine >90 µmol/L | 0 | 1/12 | 0 | 2/12 |
| Maternal adverse outcomes *(N)* | **0** | **1 (2.1)** | **1 (6.7)** | **4 (30.8)** |
| Administration of intravenous antihypertensives | 0 | 1/1 | 1/1 | 2/4 |
| Transfusion of any blood product ^¶^ | 0 | 0 | 0 | 1/4 |
| Pulmonary edema | 0 | 0 | 0 | 1/4 |
| Neonatal adverse outcomes *(N)* | **0** | **3 (6.4)** | **0** | **7 (53.8)** |
| Preterm birth <37 weeks | 0 | 3/3 | 0 | 7/7 |
| Preterm birth <32 weeks | 0 | 0 | 0 | 3/7 |
| Fetal growth restriction (birthweight <10^th^ per.) | 0 | 2/3 | 0 | 5/7 |
| NICU admission | 0 | 1/3 | 0 | 5/7 |
| Combined endpoint *(N) ** | **2 (1.6)** | **14 (29.7)** | **4 (26.7)** | **13 (100.0)** |

Data depicted as numbers (%) unless otherwise specified. Group A -/- = PCr <30 and sFlt-1/PlGF ≤38. Group B -/+ = PCr <30 and sFlt-1/PlGF >38. Group C +/- = PCr ≥30 and sFlt-1/PlGF ≤38. Group D +/+ = PCr ≥30 and sFlt-1/PlGF >38.

* Combined endpoint of patients with any preeclampsia, maternal or neonatal adverse outcomes.

Table S5. Overall pregnancy outcomes of each cohort.

| *Patients (N)* | **Group A -/-**  *N = 124* | **Group B -/+**  *N = 47* | **Group C +/-**  *N = 15* | **Group D +/+**  *N = 13* |
| --- | --- | --- | --- | --- |
| Preeclampsia diagnosis | **22 (17.7)** | **24 (51.1)** | **6 (40.0)** | **12 (92.3)** |
| *Hypertension, accompanied with one or more:* |  |  |  |  |
| Proteinuria | 17/22 | 14/24 | 6/6 | 12/12 |
| Maternal organ dysfunction | 4/22 | 5/24 | 0 | 3/12 |
| Uteroplacental dysfunction | 1/22 | 5/24 | 1/6 | 5/12 |
| Early-onset preeclampsia (<32 weeks) | 2/22 | 6/24 | 2/6 | 2/12 |
| Superimposed | 3/22 | 2/24 | 2/6 | 1/12 |
| HELLP syndrome | 0 | 2/24 | 0 | 2/12 |
| Platelet count <150x10^9^/L | 0 * | 8/24 | 2/6 | 2/12 |
| Elevated AST or ALT levels >40 U/L | 2/22 | 4/24 | 0 | 3/12 |
| Elevated LDH levels >250 U/L | 1/22 | 2/24 | 1/6 | 3/12 |
| Serum creatinine >90 µmol/L | 0 | 1/24 | 0 | 1/12 |
| Maternal adverse outcomes | **6 (4.8)** | **8 (17.0)** | **1 (6.7)** | **5 (38.5)** |
| Administration of intravenous antihypertensives | 4/6 | 5/8 | 1/1 | 2/5 |
| Hematological |  |  |  |  |
| Transfusion of any blood product * | 1/6 | 2/8 | 1/1 | 1/5 |
| Platelet count <50x10^9^/L | 0 | 0 | 0 | 0 |
| Renal failure | 0 | 0 | 0 | 0 |
| Hepatic dysfunction/hematoma/rupture | 0 | 0 | 0 | 0 |
| Cardiorespiratory |  |  |  |  |
| Myocardial ischemia/infarction | 0 | 0 | 0 | 1/5 |
| Pulmonary edema | 0 | 1/8 | 0 | 1/5 |
| Thromboembolic event | 0 | 1/8 | 0 | 1/5 |
| Central Nervous System |  |  |  |  |
| Eclampsia | 1/6 | 0 | 0 | 0 |
| Placental abruption | 0 | 1/8 | 0 | 0 |
| Maternal death | 0 | 0 | 0 | 0 |
| Neonatal adverse outcomes | **32 (25.8)** | **25 (53.2)** | **5 (33.3)** | **8 (61.5)** |
| Preterm birth <37 weeks | 17/32 | 21/25 | 4/5 | 8/8 |
| Preterm birth <32 weeks | 0 | 7/25 | 1/5 | 3/8 |
| FGR <10^th^ percentile | 14/32 | 16/25 | 0 | 6/8 |
| FGR <5^th^ percentile | 10/32 | 12 /25 | 0 | 3/8 |
| NICU admission | 13/32 | 13/25 | 4/5 | 5/8 |
| Perinatal death | 0 | 0 | 0 | 0 |
| Combined endpoint ^¶^ | **42 (33.9)** | **34 (72.3)** | **7 (46.7)** | **13 (100.0)** |

Data depicted as numbers (%) unless otherwise specified. Group A -/- = PCr <30 and sFlt-1/PlGF ≤38. Group B -/+ = PCr <30 and sFlt-1/PlGF >38. Group C +/- = PCr ≥30 and sFlt-1/PlGF ≤38. Group D +/+ = PCr ≥30 and sFlt-1/PlGF >38.

**^β^** Median (IQR) * All transfusions were administered for postpartum hemorrhage ^¶^ Combined endpoint of patients with any preeclampsia, maternal or neonatal adverse outcomes.
